# Phytochemical Profiling and Evaluation of Antioxidant and Enzyme Inhibitory Activities of Selected Endemic *Salvia* Species (Lamiaceae) From Türkiye

**DOI:** 10.1002/fsn3.72149

**Published:** 2026-07-22

**Authors:** Semih Bulut, Sümeyye Usta, Ahmet Kahraman, Mustafa Abdullah Yilmaz, Oguz Cakir, Muhammed Tilahun Muhammed

**Affiliations:** ^1^ Department of Pharmacognosy, Faculty of Pharmacy Suleyman Demirel University Isparta Turkey; ^2^ Graduate School of Health Sciences Ankara University Ankara Turkey; ^3^ Department of Molecular Biology and Genetics, Faculty of Engineering and Natural Sciences Usak University Usak Turkey; ^4^ Department of Analytical Chemistry, Faculty of Pharmacy Dicle University Diyarbakir Turkey; ^5^ Department of Nutrition and Dietetics, Atatürk Faculty of Health Sciences Dicle University Diyarbakir Turkey; ^6^ Department of Pharmaceutical Chemistry, Faculty of Pharmacy Suleyman Demirel University Isparta Turkey

**Keywords:** antidiabetic, anti‐obesity, antioxidant, LC–MS/MS, molecular docking, *Salvia*

## Abstract

*Salvia* (Lamiaceae) species have been used in food, cosmetics, and medicine for centuries. Besides their use as food, these species are potential raw material candidates for pharmaceuticals due to their rich phytochemical content. Studies on the endemic Turkish *Salvia* species examined in this study, *S. ballsiana* (Rech.f.) Hedge, *S. hedgeana* Dönmez, and *S. vermifolia* Hedge & Hub.‐Mor., are very limited. The aim of this study is to evaluate the antioxidant, antidiabetic, and anti‐obesity effects of these species and to elucidate their chemical profiles. In this context, the antidiabetic effect was determined by α‐glucosidase and α‐amylase inhibitory activity tests, and the anti‐obesity effect was determined by the pancreatic lipase inhibitory activity test. The chemical profiles of the extracts were determined by LC–MS/MS. *S. vermifolia* and *S. hedgeana* demonstrated strong α‐glucosidase inhibitory activity (IC_50_ values of 36.46 ± 1.25 and 52.89 ± 3.36 μg/mL, respectively). Among the extracts, the strongest α‐amylase and pancreatic lipase inhibitory activity was found in the *S. vermifolia* extract (IC_50_ values 68.38 ± 4.27 and 89.46 ± 6.80 μg/mL, respectively). Additionally, the reference compounds acarbose and orlistat exhibited stronger inhibitory activity than all extracts: acarbose showed IC_50_ values of 1.52 ± 0.15 and 2.99 ± 0.04 μg/mL for α‐glucosidase and α‐amylase, respectively, while orlistat demonstrated pancreatic lipase inhibition with an IC_50_ value of 11.82 ± 2.54 μg/mL. *S. vermifolia* extract demonstrated strong metal chelation capacity, ABTS and DPPH radical scavenging activity (65.50%, 70.73% and 83.80%, respectively). Rosmarinic acid was found to be major phytoconstituent of the species with the highest inhibition potency on enzymes (*S. vermifolia*). Binding potential and mode of action for rosmarinic acid were explored through molecular docking. Results that corroborated the enzymatic assay studies were obtained.

## Graphical abstract

The aerial parts extract of *Salvia vermifolia* exhibited notable inhibitory activity against α‐glucosidase and α‐amylase enzymes and demonstrated strong radical scavenging activities in the DPPH and ABTS assays.

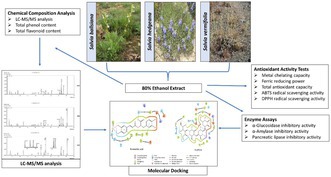

## 1 Introduction

Diabetes, a disease characterized by hyperglycemia, is projected to affect approximately 642 million people by 2040 (Ge et al. 2025). Diabetes is a major cause of morbidity worldwide, leading to macroangiopathy, neuropathy, nephropathy, retinopathy, and chronic complications (Haguet et al. 2023; Vijay et al. 2025). Dietary changes and physical activity are important factors in preventing and managing diabetes (Mlynarska et al. 2025). One of the key strategies in diabetes treatment is to inhibit the digestion and absorption of carbohydrates. Antidiabetic agents such as voglibose, acarbose, and miglitol regulate hyperglycemia by inhibiting α‐glucosidase, but these agents can cause side effects such as gastrointestinal problems, bloating, and allergies (Kashtoh and Baek 2022).

Obesity, a disease characterized by excessive fat accumulation, is seen in individuals with a body mass index above 30 kg/m^2^. Obesity and obesity‐related complications are rapidly increasing in prevalence worldwide (Williams et al. 2020). It is reported that the number of obese and overweight individuals will reach 3.8 billion by 2050, constituting more than half of the adult population (Kerr et al. 2025). Type 2 diabetes, genetic, environmental, and hormonal factors all play a role in the development of obesity. Obesity treatment includes dietary changes, physical activity, psychological support, pharmacological methods, and surgical procedures (Hawton et al. 2025). Currently, the number of drugs used in the treatment of obesity is quite limited, and orlistat, a pancreatic lipase inhibitor that reduces fat absorption, is one of them. However, as a natural consequence of the mechanism of pancreatic lipase inhibitors, side effects such as diarrhea, abdominal pain, bloating, oily stools, and stool spotting may occur (Filippatos et al. 2008).

Reducing body fat improves hyperglycemia in both diabetes and obesity. Therefore, agents that inhibit carbohydrate and lipid absorption are used in the management of diabetes and obesity (Haguet et al. 2023). However, due to the serious side effects of current α‐glucosidase and pancreatic lipase inhibitors, there is a need for new natural sources that play a role in lipid and carbohydrate metabolism (Prieto‐Rodriguez et al. 2022). In this context, interest in medicinal plants and natural resources has increased.

Lamiaceae (the mints) are the sixth largest family of flowering plants, encompassing upwards of 7000 species distributed worldwide (Harley et al. 2004). *Salvia* L., one of the largest genus in the Lamiaceae family, has traditionally been used for many years both as food and against diseases (Sharifi‐Rad et al. 2018). The genus *Salvia* has a wide distribution worldwide, primarily in the Mediterranean, America, and Africa, and is represented by approximately 1000 species (Kalnyuk et al. 2025; POWO 2025). Mexico has the most species of *Salvia* in the world, with about 300 species. Following Mexico, Türkiye possesses the second‐highest number of *Salvia* species worldwide, with approximately 100 species, nearly half of which are endemic (Celep and Doğan 2023). *Salvia* species are traditionally used to treat diabetes, coughs, ulcers, digestive problems, heart disease, and the common cold (Kalaycioglu et al. 2018). These species exhibit antioxidant, antimicrobial, anti‐inflammatory, antifungal, anticancer, antidiabetic, and anti‐obesity activities (Abdel Ghani et al. 2023; Al‐Hajj et al. 2025; Bostanci et al. 2022; Margetts et al. 2022). Extracts prepared from *Salvia* species are frequently preferred in the food, cosmetic, and pharmaceutical industries (Levaya et al. 2025; Sharifi‐Rad et al. 2018).

In vitro antioxidant activity was evaluated using extracts obtained from a large number of *Salvia* species ( *S. aethiopis* L., *S. atropatana* Bunge, *S. cyanescens* Boiss. & Balansa, *S. nemorosa* L., *S. nutans* L., *S. officinalis* L., *S. pisidica* Boiss. & Heldr. ex Benth., *S. sclarea* L., *S. syriaca* L., *S. tomentosa* Mill., *S. verticillate* L., and others) and the results have been reported in previous studies (Luca et al. 2023; Maral 2023; Moshari‐Nasirkandi et al. 2024). In the literature, studies on antidiabetic activity have been conducted across a wide range of *Salvia* species (*S. chudaei* Batt. & Trab., *S. sclarea* , *S. officinalis* , *S. lanigera* Poir, and others), focusing on the inhibition of α‐glucosidase and α‐amylase (Abd Rashed and Rathi 2021; Elshibani et al. 2025; Nemer et al. 2026; Ödemiş et al. 2026; Uğurlu et al. 2025). Similarly, previous studies, extracts obtained from numerous *Salvia* species ( *S. officinalis* , *S. dorystaechas* B.T.Drew, *S. dominica* L., and others) were evaluated for their lipase inhibition activity (Al‐Hajj et al. 2025; Ozupek et al. 2025; Zarei et al. 2025). To our knowledge, the antidiabetic and anti‐obesity effects of *S. ballsiana* (Rech.f.) Hedge, *S. hedgeana* Dönmez, and *S. vermifolia* Hedge & Hub.‐Mor., which are locally endemic to Turkey, have not been previously investigated. This study aimed to reveal the α‐glucosidase, α‐amylase, and pancreatic lipase inhibitor activities of *S. ballsiana*, *S. hedgeana*, and *S. vermifolia* for the first time. The antidiabetic effect was evaluated using α‐glucosidase and α‐amylase inhibition tests, while the anti‐obesity effect was assessed using the pancreatic lipase inhibition test. In this study, the chemical profiles of extracts were determined by Liquid Chromatography–Tandem Mass Spectrometry (LC–MS/MS) analysis, and molecular docking studies were performed.

## 2 Materials and Methods

### 2.1 Solvents and Chemicals

The names and properties of the solvents and chemicals used in this study are given below.

3,5‐dinitrosalicylic acid (≥ 98%, CAS number: 609‐99‐4), 2,2′‐azino‐bis (3‐ethylbenzothiazoline‐6‐sulfonate) (ABTS) (≥ 98%, CAS number: 30931‐67‐0), acarbose (≥ 95, CAS number: 56180‐94‐0), aluminium chloride (≥ 98%, CAS number: 7446‐70‐0), ascorbic acid (99%, CAS number: 50‐81‐7), 2,2‐diphenyl‐1‐picrylhydrazyl (DPPH) (95%, CAS number: 1898‐66‐4), ethanol (99.9%, CAS number: 64‐17‐5), ethylenediaminetetraacetic acid (EDTA) (99.4%–100.6%, CAS number: 60‐00‐4), ferrozine (97.0%, CAS number: 69898‐45‐9), Folin–Ciocalteu reagent (1.9–2.1 N, CAS number: 12111‐13‐6), gallic acid (97.5%–102.5%, CAS number: 149‐91‐7), iron (II) chloride tetrahydrate (≥ 98%, CAS number: 13478‐10‐9), lipase from porcine pancreas (≥ 125 units/mg protein, CAS number: 9001‐62‐1), maltose monohydrate (≥ 99%, CAS number: 6363‐53‐7), methanol (≥ 99.8%, CAS number: 67‐56‐1), MOPS (≥ 99.95, CAS number: 1132‐61‐2), orlistat (≥ 98%, CAS number: 96829‐58‐2), *p*‐nitrophenyl‐α‐D‐glucopyranoside (≥ 99%, CAS number: 3767‐28‐0), *p*‐nitrophenylbutyrate (≥ 98%, CAS number: 2635‐84‐9), potassium ferricyanide (III) (99%, CAS number: 13746‐66‐2), potassium persulfate (≥ 99%, CAS number: 7727‐21‐1), quercetin (≥ 95%, CAS number: 117‐39‐5), sodium acetate trihydrate (99.5%–101.0%, CAS number: 6131‐90‐4), sodium carbonate (≥ 99.5%, CAS number: 497‐19‐8), sodium potassium tartrate tetrahydrate (≥ 99%, CAS number: 6381‐59‐5), starch (CAS number: 9005‐84‐9), trichloroacetic acid (≥ 99.5%, CAS number: 76‐03‐9), trizma base (≥ 99.9, CAS number: 77‐86‐1), α‐Amylase from porcine pancreas (≥ 1000 units/mg protein, CAS: 9000‐90‐2), α‐glucosidase from *Bacillus stearothermophilus* (≥ 50 units/mg protein, CAS number: 9001‐42‐7).

### 2.2 Plant Material and Extraction

The *Salvia* species used in this study (*S. ballsiana*, *S. hedgeana*, *S. vermifolia*) were identified by Prof. Dr. Ahmet Kahraman (one of the authors). Voucher specimens are preserved in the GUL herbarium (Suleyman Demirel University). Collection information and herbarium numbers for the plants are shown in Table 1.

**TABLE 1:** Herbarium numbers and collection sites of *Salvia* species.

Plant name	Herbarium no.	Collection sites, altitude, and dates	Used part
*S. ballsiana* (Rech.f.) Hedge	GUL 94/42/30‐1	Türkiye, Adıyaman, Gerger, above Kaşyazı village, open *Quercus* scrub, about 1160 m, 10.05.2025	Aerial parts
*S. hedgeana* Dönmez	GUL 94/42/87‐1	Türkiye, Sivas, Divriği, between Divriği and Mursal, stony slopes with *Astragalus* and *Thymus* sp., 1251 m, 14.07.2025	Aerial parts
*S. vermifolia* Hedge & Hub.‐Mor.	GUL 94/42/68‐1	Türkiye, Sivas, Ulaş, between Kurtlukaya and Boğazdere, about 1.5 km to Boğazdere village, igneous rocky and stony slopes, 1519 m, 13.07.2025	Aerial parts

Plant materials were air‐dried under shade at room temperature (20°C–25°C) for 14 days under ambient humidity conditions and the aerial parts were ground into powder. Hydroalcoholic solvents used during extraction transfer many phytochemicals to the solution (Jacotet‐Navarro et al. 2018). In this context, 80% ethanol is a good solvent for transferring active phytochemicals to the solution (Bulut and Fakir 2025). The aerial parts (10 g) were extracted using the maceration method with 80% ethanol (200 mL) and left to stand for 16 h (Bulut and Fakir 2025). At the end of the period, the extract was filtered and concentrated using a rotary evaporator (Heidolph). This procedure was repeated three times.

### 2.3 Chemical Composition Analysis

#### 2.3.1 Total Phenol Content

To determine the total phenol content of *Salvia* extracts, 10% Folin–Ciocalteu and 7.5% sodium carbonate solutions were prepared. Then, Folin–Ciocalteu and sodium carbonate solutions were added to the *Salvia* extracts (1 mg/mL), and the mixture was reacted in a dark cabinet at 25°C for 30 min. The absorbance of the final mixture was measured at 735 nm. The total phenolic contents of the extracts were expressed as mg gallic acid equivalent (GAE)/g extract (Zongo et al. 2010).

#### 2.3.2 Total Flavonoid Content

After adding ethanol (95%), sodium acetate (1 M), and AlCl_3_ (10%) to *Salvia* extracts (1 mg/mL), the total volume of the mixture was brought to 1000 μL with water. The final mixture was then reacted at 25°C for 30 min, and the absorbance was measured at 415 nm at the end of this period. The total flavonoid contents of the extracts were expressed as mg quercetin equivalent (QE)/g extract (Kosalec et al. 2004).

#### 2.3.3 LC–MS/MS Analysis

This analysis was performed using a validated and verified methodology (Yilmaz 2020). For the quantitative evaluation of phytochemicals, a mass spectrometer and ultrahigh performance liquid chromatography (Shimadzu‐Nexera) were used. During the analysis, an Agilent Poroshell column (120 EC–C18, 2.7 μm, 150 mm × 2.1 mm) was preferred. Analysis parameters: column temperature—40°C, gradient: eluent Y (H_2_O + ammonium formate [5 mM] + HCOOH [0.1%]) and eluent Z (CH_3_OH + ammonium formate [5 mM] + HCOOH [0.1%]), injection volume—5 μL, flow rate—0.5 mL/min. Gradient flow parameters: 20%–100% Z (0–25 min), 100% Z (25–35 min), 20% Z (35–45 min). A Shimadzu LCMS–8040 tandem mass spectrometer equipped with an electrospray ionization source was used during the analysis. Mass spectrometric detection was performed in multiple reaction monitoring mode, and the multiple reaction monitoring transitions, precursor/product ions, retention times, and collision energies of each analyte were optimized individually using authentic reference standards, as previously described by Yilmaz (2020). Compound identification was accomplished by comparing the retention times and characteristic multiple reaction monitoring ion transitions of the detected peaks with those of authentic standards analyzed under identical experimental conditions. The mass spectrometry parameters: drying gas (N_2_) flow, 15 L/min; interface temperature, 350°C; DL temperature, 250°C; heat block temperature, 400°C and nebulizing gas (N_2_) flow, 3 L/min. Analytical method validation parameters for standard phytochemicals are shown in Table S1 (Yilmaz 2020).

### 2.4 Antioxidant Activity Tests

#### 2.4.1 Metal Chelating Capacity

To determine antioxidant activity, FeCl_2_ (2 mM) and ferrozine (5 mM) solutions were prepared. *Salvia* extracts were mixed with FeCl_2_ (2 mM) and left to stand at 25°C for 5 min. Ferrozine (5 mM) was poured onto the final mixture and after reacting at 25°C for 10 min, the absorbance was measured at 562 nm. In this test, EDTA was used as the reference substance (Dinis et al. 1994). The activity was calculated using the following Equation (1).

(1) $$\text{Acitivty}\%=\frac{\text{Absorbance control}-\text{Absorbance extract}}{\text{Absorbance control}}\;\times 100$$

#### 2.4.2 Ferric Reducing Power

Phosphate buffer (pH = 7.2, 0.1 mol/L) was transferred onto the *Salvia* extracts. K_3_Fe(CN)_6_ (1%) was added to the mixture and the mixture was reacted at 37°C for 60 min. At the end of the reaction, 10% trichloroacetic acid was added to the mixture and the absorbance was measured at 700 nm. After absorbance measurement, FeCl_3_ (0.1%) was added to the extract and absorbance was measured again at 700 nm. The reduction power was calculated by taking the difference between the absorbance values read at 700 nm (Orhan et al. 2017).

#### 2.4.3 Total Antioxidant Capacity

*Salvia* extracts (1 mg/mL) were mixed with molybdate reagent (1000 μL), and the extract‐molybdate reagent mixture was reacted at 95°C for 90 min. The mixture was then cooled, and the absorbance was measured at 695 nm. Antioxidant capacity was expressed as mg ascorbic acid equivalent (AAE)/g extract (Prieto et al. 1999).

#### 2.4.4 ABTS Radical Scavenging Activity

To determine antioxidant activity, a solution of 7 mM ABTS and 2.45 mM potassium persulfate was mixed, and the mixture was left to stand in a colored bottle in the dark for 12 h. This mixture was then dissolved in phosphate buffer (pH = 7.4) to form dilute ABTS. The diluted ABTS (1000 μL) and *Salvia* extracts were mixed and reacted for 6 min. At the end of the reaction, the absorbance of the mixture was measured at 734 nm. In this test, gallic acid was used as the reference substance (Orhan et al. 2017). The activity was calculated using the following Equation (2).

(2) $$\text{Acitivty}\%=\frac{\text{Absorbance control}-\text{Absorbance extract}}{\text{Absorbance control}}\;\times 100$$

#### 2.4.5 DPPH Radical Scavenging Activity

To determine antioxidant activity, a first 1 mM DPPH reagent was prepared. *Salvia* extracts and DPPH reagent were mixed to obtain a final volume of 100 μL. After the mixture was left to stand in a dark cabinet for 30 min, the absorbance was measured at 520 nm. In this test, ascorbic acid was used as the reference substance (Jung et al. 2011). The activity was calculated using the following Equation (3).

(3) $$\text{Acitivty}\%=\frac{\text{Absorbance control}-\text{Absorbance extract}}{\text{Absorbance control}}\;\times 100$$

### 2.5 Enzyme Assays

#### 2.5.1 α‐Glucosidase Inhibitory Activity

Phosphate buffer (pH = 6.5, 0.5 M) was added to the *Salvia* extracts. Then, α‐glucosidase type IV enzyme (in phosphate buffer) was added to the mixture, and the mixture was reacted at 37°C for 15 min. After the reaction, 20 mM *p*‐nitrophenyl‐α‐D‐glucopyranoside was added to the mixture as a substrate and incubated at 37°C for 35 min. The absorbance of the extracts was then read at 405 nm. In this test, acarbose was used as the reference substance, and all procedures were applied to acarbose (Lam et al. 2008).

#### 2.5.2 α‐Amylase Inhibitory Activity

Phosphate buffer (pH = 6.9) and α‐amylase type I‐A enzyme were added to the *Salvia* extracts (in phosphate buffer), and the mixture was left to stand at 25°C for 5 min. Then, 2.5% potato starch solution was poured over the mixture and left to react for 15 min. At the end of the reaction, a color reagent (sodium potassium tartrate [5.31 M] + 3,5‐dinitrosalicylic acid [96 mM]) was added to the mixture and the mixture was left to stand at 80°C for 40 min. Then, cold water was added to the mixture and the absorbance was measured at 540 nm. Activity was calculated by creating a maltose calibration curve. In this test, acarbose was used as the reference substance, and all procedures were applied to acarbose (Ali et al. 2006).

#### 2.5.3 Pancreatic Lipase Inhibitory Activity

Tris–HCl buffer (Tris–HCl [100 mM] and CaCl_2_ [5 mM]) and pancreatic lipase enzyme Type II were transferred onto *Salvia* extracts. Then, pH = 6.8 MOPS buffer (MOPS [10 mM], EDTA [1 mM]) was added to the mixture and the mixture was reacted at 37°C for 15 min. At the end of the reaction, *p*‐nitrophenylbutyrate (10 mM) was transferred to the mixture as a substrate and incubated at 37°C for 30 min. Then the absorbance was measured at 405 nm. In this test, orlistat was used as the reference substance, and all procedures were applied to orlistat (Lee et al. 2012).

### 2.6 Molecular Docking

Crystal structures of α‐amylase, α‐glucosidase, and pancreatic lipase were downloaded from the protein data bank (PDB). Enzyme structures of human α‐amylase with PDB code of 2QV4, α‐glucosidase with PDB code of 8YIE, and pancreatic lipase with PDB code of 2PPL were used for docking analysis. Human α‐amylase and α‐glucosidase structures beard co‐crystalline acarbose (Maurus et al. 2008; Saburi et al. 2024). The co‐crystalline acarbose structures were used for validating docking procedures. Rosmarinic acid and ligand structures were retrieved from the PubChem database. Compound structures were prepared for docking procedure by using LigPrep module of Maestro 14.5 program. Possible protonation states were enacted at pH value of 7.00 ± 0.50 with Epik. Similarly, enzyme structures were prepared for docking by adding missing side chains, deleting water, and doing optimization as well as minimization using OPLS‐2005 force field. Determination of protonation states and assignment of charges were also performed. Grid box specifications for enzyme structures were done using receptor grid generation module. Docking procedure was performed by using ligand docking module of Schrödinger release 2025‐3 (Faydali et al. 2026; Naheed et al. 2025).

### 2.7 Statistical Analysis

The analyses for this study were repeated three times. Analysis results are presented as mean ± standard deviation (SD). In the comparison of extracts across contents, a one‐way ANOVA test was employed with Tukey HSD post hoc test, and in activity groups, a one‐way ANOVA test was employed with Dunnett *t* post hoc test. Normality was assessed using the Shapiro–Wilk test and homogeneity of variances was evaluated using Levene's test. A mixed design of two‐way ANOVA was used to compare the extracts between dose groups with Dunnett *t* post hoc test for reference values. The Spearman's Rho correlation test was performed to determine the relations between the extract measurements between content and activity groups. A *p* < 0.05 value was considered statistically significant. IC_50_ values were calculated using extract dilutions ranging from 5 to 200 μg/mL, while dilutions of 3.125–200 μg/mL were used for orlistat and 0.000625–200 μg/mL for acarbose.

## 3 Results

The comparisons between the extracts in different contents reveal that *S. vermifolia* is the most potent species analyzed, exhibiting the highest total phenolic content (80.35 mg GAE/g) and the strongest total antioxidant capacity (187.13 mg AAE/g). While *S. ballsiana* and *S. vermifolia* show comparable levels of total flavonoids, *S. hedgeana* lags significantly behind in this category. Statistically, the very low *p* values (*p* < 0.05) across all parameters confirm that the differences between these extracts are highly significant (Table 2).

**TABLE 2:** Results of total phenol, total flavonoid content, and total antioxidant capacity of the extracts.

Extract	Total flavonoid content (mg QE/g extract ± SD)	Total phenolic content (mg GAE/g extract ± SD)	Total antioxidant capacity (mg AAE/g extract ± SD)
*S. ballsiana*	47.18 ± 2.12^a^	61.93 ± 1.73^a,b^	100.74 ± 2.58^a,b^
*S. hedgeana*	32.61 ± 1.63^a,b^	69.51 ± 1.64^a,c^	157.25 ± 0.97^a,c^
*S. vermifolia*	48.36 ± 1.47^b^	80.35 ± 0.78^b,c^	187.13 ± 3.94^b,c^
*p* value	0.038*	0.003*	0.002*

*Note:* Same superscript letters (a–c) denote the significant pairwise comparisons according to Tukey HSD post hoc test.

Abbreviations: AAE, ascorbic acid equivalent; GAE, gallic acid equivalent; QE, quercetin equivalent.

* Significant at 0.05 level according to ANOVA.

**FIGURE 1:**
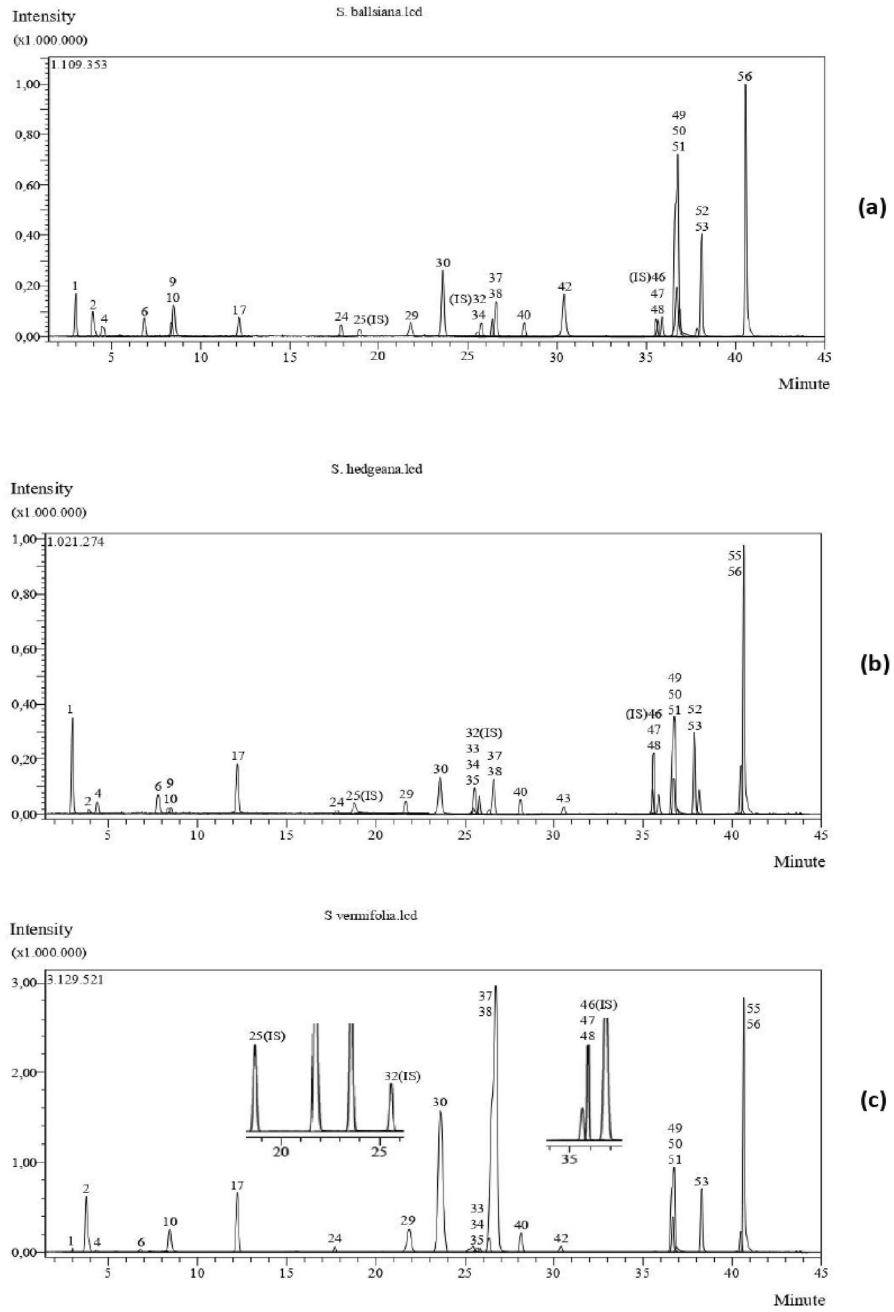
LC–MS/MS chromatogram of *S. ballsiana* (a), *S. hedgeana* (b), and *S. vermifolia* (c).

**FIGURE 2:**
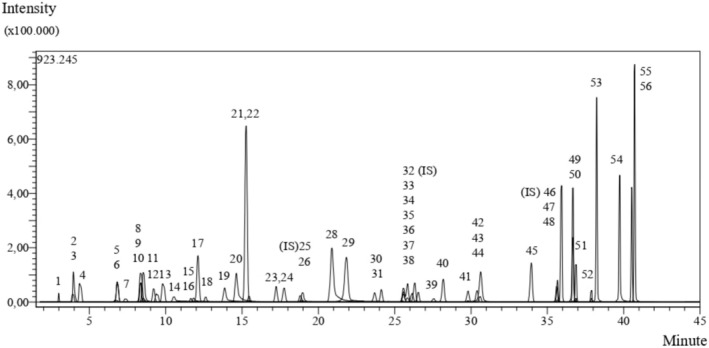
LC–MS/MS chromatogram of standard compounds.

LC–MS/MS chromatograms of standard compounds and extracts are given in Figures 1 and 2. When the chemical profiles of the extracts were examined by LC–MS/MS analysis, it was determined that *S. vermifolia* contained the most abundant rosmarinic acid, cynaroside, fumaric acid, and acacetin (30.633, 23.287, 18.778, and 12.814 mg/g extract, respectively). Although acacetin and luteolin were found to be the most abundant in *S. ballsiana* extract, these results were considerably lower than those of *S. vermifolia* (4.57 and 4.355 mg/g extract, respectively). Rosmarinic acid and acacetin were found to be the most abundant in *S. hedgeana*, but similarly, these results were lower than those of *S. vermifolia* (5.48 and 4.483, respectively). The LC–MS/MS analysis results of the extracts are shown in detail in Table 3. Furthermore, validation parameters for the standard compounds are given in Table S1.

**TABLE 3:** Chemical composition results of *Salvia* extracts.

No.	Compounds	*S. ballsiana* (mg analyte/g extract)	*S. hedgeana* (mg analyte/g extract)	*S. vermifolia* (mg analyte/g extract)
1	Quinic acid	1.046	0.684	0.23
2	Fumaric acid	0.702	0.062	18.778
3	Aconitic acid	N.D.	N.D.	0.018
4	Gallic acid	0.017	0.011	0.014
5	Epigallocatechin	N.D.	N.D.	N.D.
6	Protocatechuic acid	0.155	0.234	0.596
7	Catechin	N.D.	N.D.	N.D.
8	Gentisic acid	N.D.	N.D.	N.D.
9	Chlorogenic acid	0.039	0.017	N.D.
10	Protocatechuic aldehyde	0.254	0.289	0.994
11	Tannic acid	N.D.	N.D.	N.D.
12	Epigallocatechin gallate	N.D.	N.D.	N.D.
13	Cynarin	N.D.	N.D.	N.D.
14	4‐OH Benzoic acid	N.D.	N.D.	N.D.
15	Epicatechin	N.D.	N.D.	N.D.
16	Vanilic acid	N.D.	N.D.	N.D.
17	Caffeic acid	0.484	0.795	1.884
18	Syringic acid	N.D.	N.D.	N.D.
19	Vanillin	N.D.	N.D.	N.D.
20	Syringic aldehyde	N.D.	N.D.	N.D.
21	Daidzin	N.D.	N.D.	N.D.
22	Epicatechin gallate	N.D.	N.D.	N.D.
23	Piceid	N.D.	N.D.	N.D.
24	*p*‐Coumaric acid	0.105	0.094	0.523
25	Ferulic acid‐D3‐IS^h^	N.A.	N.A.	N.A.
26	Ferulic acid	N.D.	N.D.	N.D.
27	Sinapic acid	N.D.	N.D.	N.D.
28	Coumarin	N.D.	N.D.	N.D.
29	Salicylic acid	0.069	0.136	0.433
30	Cyranoside	3.616	0.624	23.287
31	Miquelianin	N.D.	N.D.	N.D.
32	Rutin‐D3‐IS	N.A.	N.A.	N.A.
33	Rutin	N.D.	0.24	0.048
34	Isoquercitrin	0.859	0.096	0.445
35	Hesperidin	N.D.	1.193	0.214
36	*O*‐Coumaric acid	N.D.	N.D.	N.D.
37	Genistin	0.838	0.314	6.446
38	Rosmarinic acid	3.197	5.48	30.633
39	Ellagic acid	N.D.	N.D.	N.D.
40	Cosmosiin	0.68	0.262	5.301
41	Quercitrin	N.D.	N.D.	N.D.
42	Astragalin	3.642	N.D.	0.077
43	Nicotiflorin	N.D.	0.138	N.D.
44	Fisetin	N.D.	N.D.	N.D.
45	Daidzein	N.D.	N.D.	N.D.
46	Quercetin‐D3‐IS	N.A.	N.A.	N.A.
47	Quercetin	0.13	0.128	0.042
48	Naringenin	0.048	0.151	0.148
49	Hesperetin	0.014	0.024	0.024
50	Luteolin	4.355	2.126	6.198
51	Genistein	0.006	0.003	0.01
52	Kaempferol	0.051	0.083	N.D.
53	Apigenin	1.171	0.858	2.069
54	Amentoflavone	N.D.	N.D.	N.D.
55	Chrysin	N.D.	0.023	0.005
56	Acacetin	4.57	4.483	12.814

Abbreviations: N.A., not applicable; N.D., not detected.

The DPPH radical scavenging activities of the extracts ranged from 20.11% to 83.80%. At all concentrations, DPPH radical scavenging activity was found to be strongest in the *S. vermifolia* extract. Extracts of *S. hedgeana*, *S. ballsiana*, and *S. vermifolia* exhibited moderate metal chelation activity (47.09%, 56.68%, and 65.50%, respectively). *S. hedgeana* and *S. ballsiana* showed moderate ABTS radical scavenging activity, while *S. vermifolia* extract exhibited strong ABTS radical scavenging activity (42.40%, 50.44%, and 70.73%, respectively). Similarly, although the highest ferric reducing power absorbance value was observed in the *S. vermifolia* extract, this value was found to be lower than that of quercetin. Statistically, the two‐way ANOVA confirms that both the concentration and the specific extract type have a significant impact on activity (*p* < 0.05). The antioxidant activity results of the extracts are shown in Table 4.

**TABLE 4:** Antioxidant activity results of *Salvia* extracts.

Samples	μg/mL	DPPH radical scavenging activity inhibition % ± SD	Metal chelating capacity % ± SD	ABTS radical scavenging activity inhibition % ± SD	Ferric reducing power absorbance ± SD
*S. ballsiana*	250	—	40.46 ± 0.77	27.17 ± 1.13	0.175 ± 0.02
	500	20.11 ± 3.97	45.74 ± 1.61	32.62 ± 2.32	0.221 ± 0.03
	1000	34.90 ± 1.56	53.09 ± 2.21	46.79 ± 3.21	0.432 ± 0.04
	2000	47.65 ± 1.08	56.68 ± 1.91	50.44 ± 3.03	0.801 ± 0.02
	*p*_dose_	< 0.001*	< 0.001*	< 0.001*	< 0.001*
*S. hedgeana*	250	—	32.36 ± 2.02	16.27 ± 0.52	0.210 ± 0.02
	500	38.11 ± 2.02	43.85 ± 1.30	27.21 ± 0.69	0.280 ± 0.03
	1000	62.91 ± 2.61	46.74 ± 1.61	40.25 ± 2.36	0.571 ± 0.02
	2000	71.87 ± 0.79	47.09 ± 0.15	42.40 ± 3.48	0.870 ± 0.02
	*p*_dose_	< 0.001*	< 0.001*	< 0.001*	< 0.001*
*S. vermifolia*	250	50.78 ± 3.91	47.66 ± 0.78	31.53 ± 1.33	0.275 ± 0.01
	500	79.97 ± 0.75	51.47 ± 0.72	35.30 ± 1.45	0.467 ± 0.02
	1000	80.67 ± 1.70	60.29 ± 0.22	51.66 ± 2.57	1.021 ± 0.01
	2000	83.80 ± 0.70	65.50 ± 0.19	70.73 ± 0.44	1.579 ± 0.03
	*p*_dose_	0.014*	< 0.001*	< 0.001*	< 0.001*
Reference	250	84.62 ± 0.23^a^	99.51 ± 0.43^b^	98.03 ± 0.46^c^	3.013 ± 0.03^d^
	500	85.44 ± 0.26^a^	99.91 ± 0.26^b^	98.18 ± 0.36^c^	3.129 ± 0.02^d^
	1000	86.58 ± 2.95^a^	99.86 ± 0.21^b^	99.09 ± 0.22^c^	3.180 ± 0.04^d^
	2000	89.67 ± 0.35^a^	99.95 ± 0.11^b^	99.47 ± 0.08^c^	3.281 ± 0.00^d^
*p* value_extracts_		0.036*	< 0.001*	< 0.001*	< 0.001*

Abbreviation: SD, standard deviation.

^a^ Ascorbic acid.

^b^ EDTA.

^c^ Gallic acid.

^d^ Quercetin.

* Significant at 0.05 level according to two‐way ANOVA with mixed design, all pairwise dose comparisons are significant by Tukey HSD post hoc test, all pairwise extract comparisons with reference values are significant by Dunnett *t* post hoc test.

The antidiabetic potential of *Salvia* extracts was evaluated using α‐glucosidase and α‐amylase inhibition methods. When the results were examined, *S. vermifolia* and *S. hedgeana* showed strong α‐glucosidase inhibitory activity (IC_50_ values were 36.46 ± 1.25 and 52.89 ± 3.36 μg/mL, respectively). Although all three extracts inhibited the α‐amylase enzyme, the strongest inhibition was observed in the *S. vermifolia* extract. The pancreatic lipase inhibitory activities of the extracts ranged from 38.57% to 63.96%. Similar to other enzyme inhibition tests, the strongest pancreatic lipase inhibitory activity was found in *S. vermifolia* extract (IC_50_ = 89.46 ± 6.80 μg/mL). The statistical analysis confirms these findings, as the *p* values (*p* < 0.003) show that the differences in potency between the extracts are highly significant. However, it is important to note that the reference drugs remain vastly superior to the plant extracts, with IC_50_ values that are many times lower than those of the most potent extract. The enzyme inhibition results of *Salvia* extracts are shown in Table 5.

**TABLE 5:** Enzyme inhibition results of *Salvia* extracts.

Samples	Inhibition % ± SD at 200 μg/mL^a^ (IC_50_: μg/mL ± SD)		
	α‐Glucosidase	α‐Amylase	Pancreatic lipase
*S. ballsiana*	58.77 ± 3.00 (IC_50_ = 129.06 ± 8.48)	35.30 ± 2.21	41.25 ± 3.48
*S. hedgeana*	72.99 ± 2.73 (IC_50_ = 52.89 ± 3.36)	31.95 ± 1.52	38.57 ± 1.72
*S. vermifolia*	75.15 ± 1.53 (IC_50_ = 36.46 ± 1.25)	66.28 ± 0.88 (IC_50_ = 68.38 ± 4.27)	63.96 ± 2.01 (IC_50_ = 89.46 ± 6.80)
Reference	99.91 ± 0.15^b^ (IC_50_ = 1.52 ± 0.15^b^)	96.55 ± 0.27^b^ (IC_50_ = 2.99 ± 0.04^b^)	73.33 ± 1.42^c^ (IC_50_ = 11.82 ± 2.54^c^)
*p* value	0.003*	< 0.001*	0.001*

Abbreviation: SD, standard deviation.

^a^ Final concentration.

^b^ Acarbose.

^c^ Orlistat.

* Significant at 0.05 level according to ANOVA, all pairwise comparisons between reference and other salvia extract groups are significant according to Dunnett *t* post hoc test.

Moreover, the correlations between the values of extracts in content and activity were assessed using Spearman's Rho. The measurements of *S. ballsiana* showed no significant correlation between content and activity (*R* = −0.483; *p* = 0.187), and *S. hedgeana* showed a similar correlation (*R* = −0.469; *p* = 0.203). However, *S. vermifolia* showed a very strong correlation between content and activity measurements (*R* = −0.937; *p* < 0.001).

Binding potential and mode of interaction for major phytoconstituent of the *Salvia* species with the highest activity on all three enzymes (*S. vermifolia*), rosmarinic acid, were explored through molecular docking. Docking procedure validation was performed prior to docking of rosmarinic acid. For this purpose, co‐crystalline acarbose structures were re‐docked on respective crystal α‐amylase and α‐glucosidase structures. Then, root mean square deviation (RMSD) value between crystal and docked acarbose was computed. RMSD values for acarbose inside α‐amylase and α‐glucosidase were calculated to be 1.4769 and 1.3748 Å, respectively.

Docking of rosmarinic acid was performed and compared to standard inhibitors for α‐amylase, α‐glucosidase, and pancreatic lipase. Binding energy related values for rosmarinic acid were found to be higher in comparison to acarbose in its interaction with α‐amylase and α‐glucosidase structures. On the other hand, binding energy related values for rosmarinic acid were lower in comparison to orlistat in its interaction with pancreatic lipase (Table 6).

**TABLE 6:** Binding energy related values of rosmarinic acid and standard drugs in their interactions with the α‐amylase, α‐glucosidase, and pancreatic lipase structures (in kcal/mol).

Compounds	Target	Docking score	Glide energy	Glide gscore	Glide emodel
Rosmarinic acid	2QV4	−4.83	−42.18	−4.83	−49.76
Acarbose	2QV4	−6.87	−47.27	−7.20	−48.43
Rosmarinic acid	8YIE	−5.71	−40.38	−5.71	−52.72
Acarbose	8YIE	−6.53	−57.96	−6.87	−78.65
Rosmarinic acid	2PPL	−5.02	−40.52	−5.02	−53.08
Orlistat	2PPL	−4.01	−36.92	−4.23	−37.22

**FIGURE 3:**
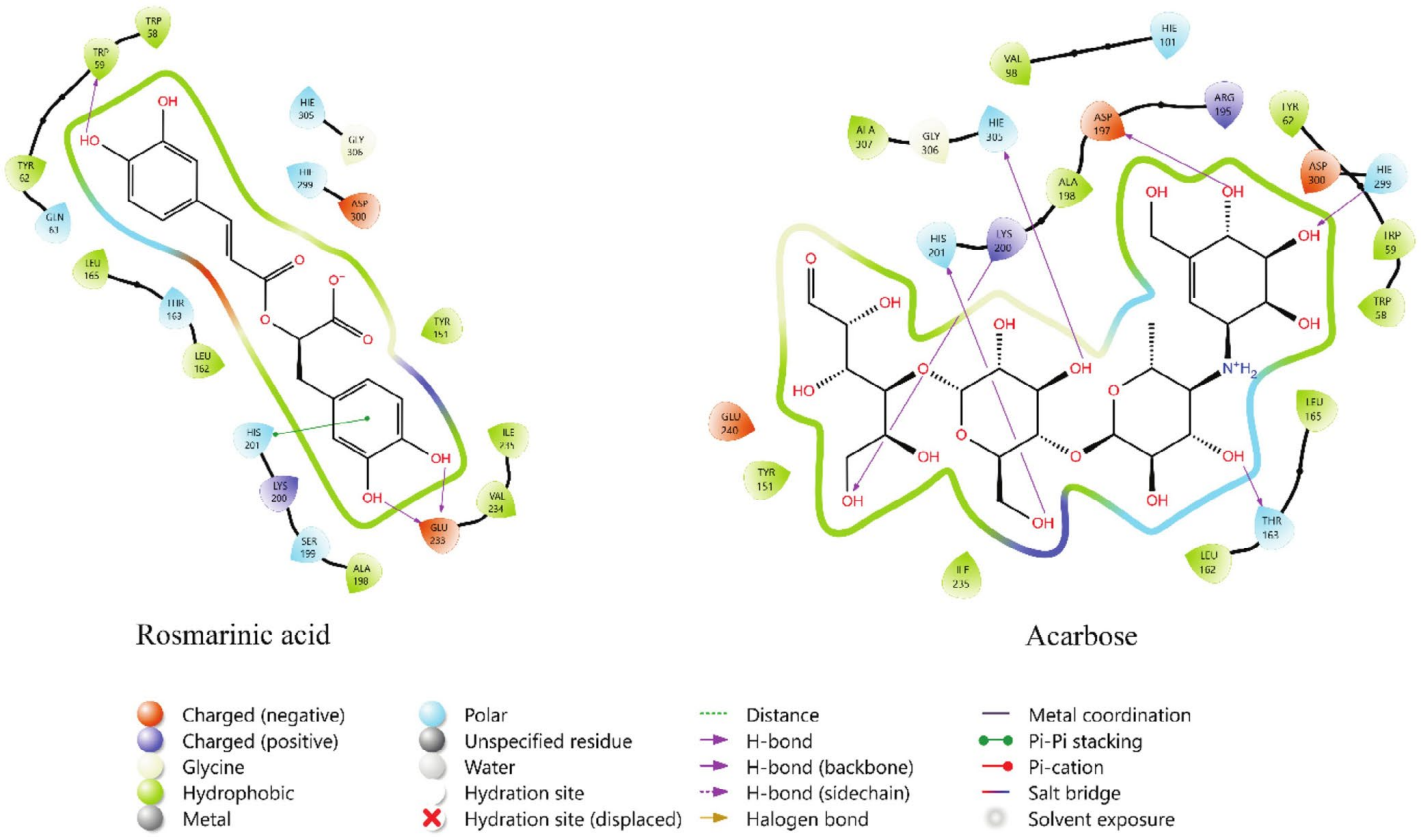
Binding profiles of rosmarinic acid and acarbose in their interaction with α‐amylase structure (2QV4).

Interactions of rosmarinic acid and acarbose to α‐amylase (2QV4) were determined through docking. Rosmarinic acid formed three H‐bonds via Trp59 and Glu233 (x2). In addition, it had pi‐pi stacking interaction via His201 (Figure 3). Similarly, acarbose formed six H‐bonds with the α‐amylase structure via the involvement of Thr163, Asp197, Lys200, His201, His299, and His305 residues (Figure 3).

**FIGURE 4:**
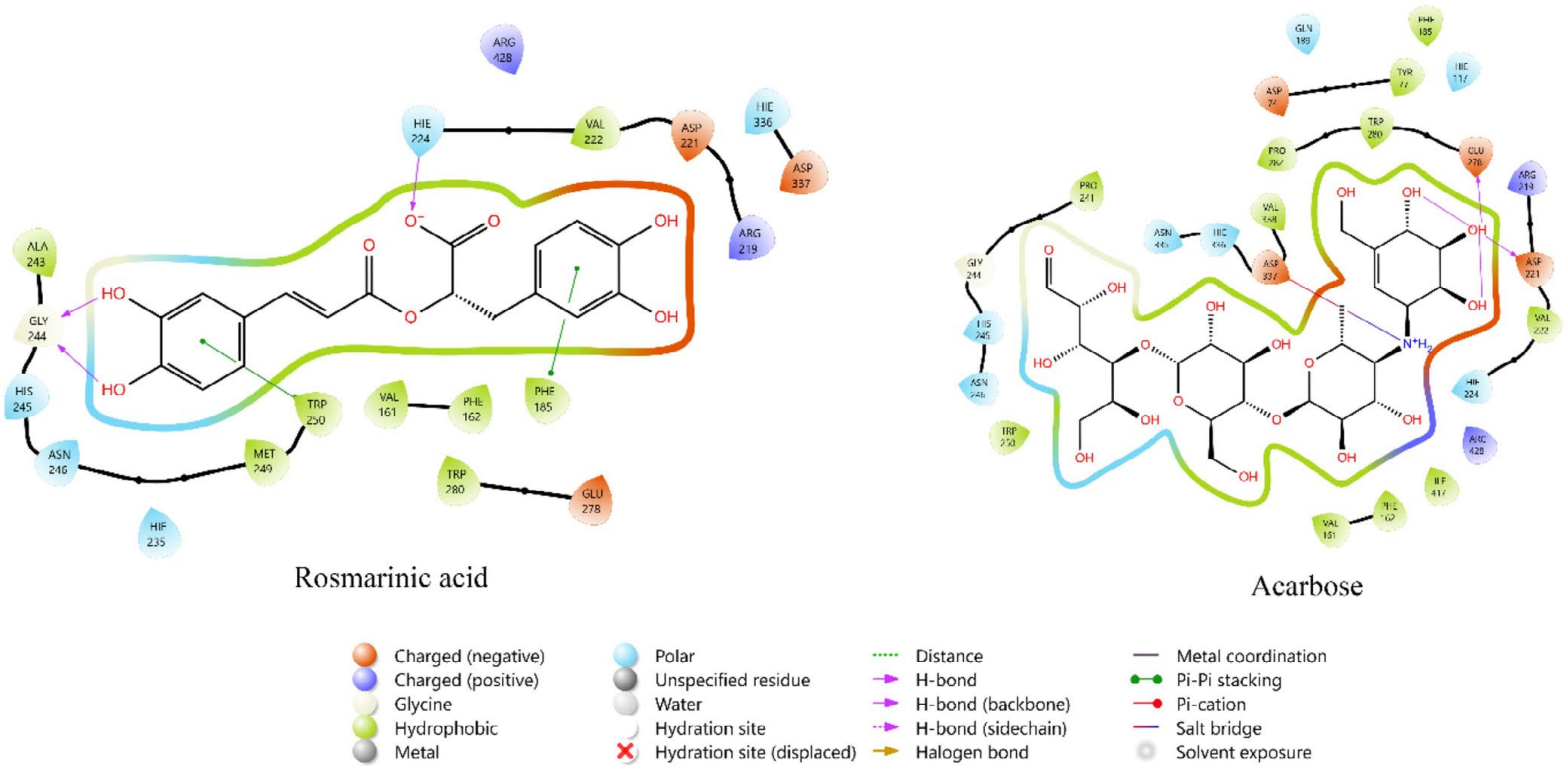
Binding profiles of rosmarinic acid and acarbose in their interaction with α‐glucosidase structure (8YIE).

Rosmarinic acid and acarbose interacted with α‐glucosidase (8YIE) structure at various levels. Rosmarinic formed H‐bonds via involvement of His224 and Gly244 (x2) with the α‐glucosidase structure. In addition, it formed pi‐pi stacking interactions via Phe185 and Trp250 (Figure 4). On the other hand, acarbose formed three H‐bonds (Asp221, Glu278 (x2)) and a salt bridge interaction with Asp337 in its interactions with the α‐glucosidase structure (Figure 4).

**FIGURE 5:**
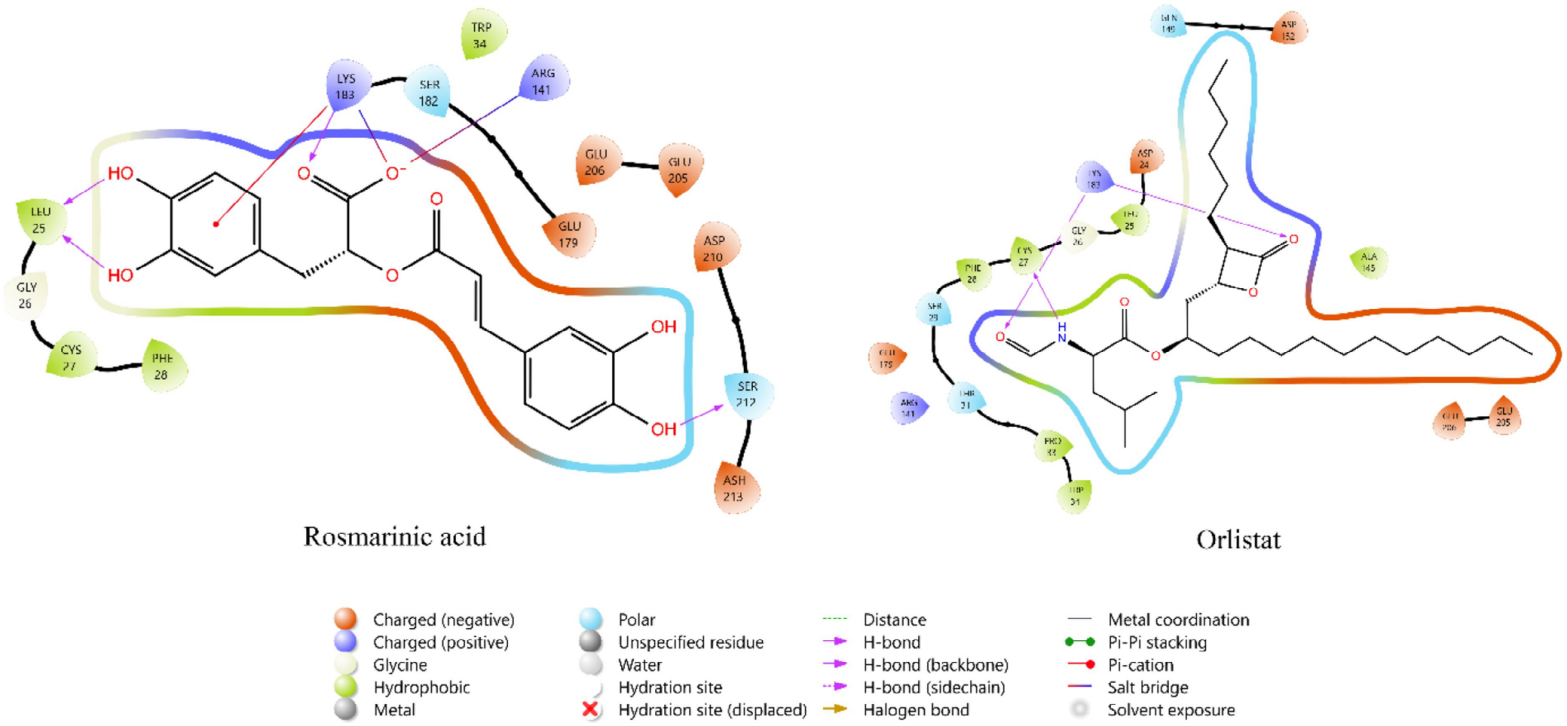
Binding profiles of rosmarinic acid and orlistat in their interaction with pancreatic lipase structure (2PPL).

Rosmarinic acid and orlistat interacted with pancreatic lipase structure (2PPL) at different levels. Rosmarinic acid formed four H‐bonds (Leu25(x2), Lys183, Ser212), a salt bridge (Arg141, Lys183), and pi‐pi stacking (Lys183) interactions with the pancreatic lipase structure. On the other hand, orlistat formed three H‐bonds (Cys27, Lys183(x2)) with the pancreatic lipase structure (Figure 5).

## 4 Discussion

In this study, we used numerous mechanisms to determine the antioxidant activity of the extracts. These mechanisms include ABTS, DPPH, metal chelation, total antioxidant capacity, and ferric reducing power tests. In this context, the antioxidant activity results of *S. vermifolia* extract were notable. *S. vermifolia* extract demonstrated strong ABTS and DPPH radical scavenging activity. The antioxidant activity of *S. ballsiana* and *S. hedgeana* extracts was evaluated as moderate. *Salvia* species have been reported to exhibit significant antioxidant activity due to their radical scavenging effects (Henriquez et al. 2023). In a study conducted by Moshari‐Nasirkandi et al. evaluating the antioxidant effects of 20 *Salvia* species, the DPPH radical scavenging activity results ranged between 4.58 and 68.93 μg AAE/mL, while the ferric reducing power analysis results varied between 64.28 and 1071.79 μmol Fe^2+^/g. The strongest DPPH radical scavenging activity and ferric reducing power activity were observed in the *S. limbata* C.A.Mey. extract (Moshari‐Nasirkandi et al. 2024). Similar to the study by Moshari‐Nasirkandi et al., in our study it was also found that the extract with the strongest DPPH scavenging activity also had the strongest ferric reducing power activity. This suggests that *S. vermifolia* exhibits free radical scavenging effect through different mechanisms. Similar to our study, *S. hedgeana* has been reported to possess DPPH radical scavenging activity and ferrous ferric‐chelating capacity (Şenol et al. 2010). In a study conducted by Nilofar et al. (2024) reported that *S. microstegia* Boiss. & Balansa exhibited strong DPPH, ABTS, and ferric reducing power activities. In this study, unlike our work, since the volatile oil of the plant was investigated, the strong activity was attributed to the sesquiterpene content. Similarly, another study reported that the high flavonoid and terpenoid content of *S. prattii* Hemsl. significantly contributes to its antioxidant activity (Xia et al. 2024). Özüpek et al. evaluated the antioxidant effect of organically grown *S. officinalis* and *S. triloba* L.f. The results of the study showed that the DPPH radical scavenging effects of the extracts ranged between 56.98% and 79.04%. At the same time, the ferric reducing power absorbance values of these extracts ranged from 1.293 to 3.392 ± 0.01 (Özüpek et al. 2023). Özüpek et al. found stronger ferric reducing power and DPPH radical scavenging activity compared to our study. This may be related to the fact that the chemical content of plants grown through organic farming is more controllable. When the individual compound of the extracts were evaluated, it was observed that the amounts of fumaric acid, protocatechuic acid, protocatechuic aldehyde, caffeic acid, *p*‐coumaric acid, salicylic acid, genistin, rosmarinic acid, cosmosiin, luteolin, apigenin, and acacetin were higher in the *S. vermifolia* extract compared to the other extracts. Fumaric acid found in plants exhibits antioxidant activity due to its strong free radical scavenging properties (Jadeja et al. 2020; Kaur et al. 2020). Protocatechuic acid and protocatechuic aldehyde exhibit antioxidant activities both by scavenging free radicals and by enhancing the activity of endogenous antioxidant enzymes (Amic et al. 2025; Coksu et al. 2025; Zhang et al. 2021). Caffeic acid, *p*‐coumaric acid, and salicylic acid reduce oxidative stress thanks to their radical scavenging effects (Halpani and Mishra 2024; Purushothaman et al. 2022; Singh 2023). Genistin exhibits significant antioxidant activity thanks to its hydrogen atom and electron transfer mechanism (Qi et al. 2023). Recent studies have indicated that rosmarinic acid found in plants shows antioxidant effects by scavenging free radicals and chelating metals (Boufetacha et al. 2024; Kowalczyk et al. 2024; Vo et al. 2023). Similar to other compounds, cosmosiin, luteolin, and apigenin are important antioxidant compounds due to their DPPH and ABTS radical scavenging activity and ferric reducing power (Lalfakawmi et al. 2023; Tian et al. 2021). Acacetin found in plants increases heme oxygenase 1 expression while suppressing the production of reactive oxygen species (Zhang et al. 2025). In this context, the strong activity of *S. vermifolia* extract observed in all tested antioxidant tests can be explained by its rich chemical composition. These compounds may also have exhibited a synergistic effect, enhancing antioxidant activity.

In this study, the total phenol and total flavonoid content results of *Salvia* extracts were found to be similar. When the results were examined, *S. vermifolia* was found to have the highest total phenol and total flavonoid content (80.35 ± 0.78 mg GAE/g extract and 48.36 ± 1.47 mg QE/g extract, respectively). The total phenol and total flavonoid content of the flower parts of *Salvia sclarea* were reported as 26.4 ± 7.78 mg GAE/g extract and 10.44 ± 0.21 mg QE/g extract, respectively (Torunoglu et al. 2024). In a study conducted in Türkiye, the total phenol content of the aerial parts of *S. aethiopis* , *S. candidissima* Vahl, *S. limbata* , *S. microstegia*, *S. nemorosa* , *S. pachystachys* Trautv., *S. verticillate* L., and *S. virgata* Jacq., was found to ranged between 50.3 and 167.1 mg GAE/g extract (Tosun et al. 2009). The total phenol content of leaf, root, stem, and flower extracts of *S. atropatana*, *S. frigida* Boiss., *S. macrosiphon* Boiss., *S. nemorosa* , *S. officinalis* , *S. sclarea* , *S. syriaca* , and *S. virgata* collected from Iran ranged from 1.5 to 40.6 mg GAE/g extract (Esmaeili et al. 2022). The differences between the results of the current study and those of other studies may be due to geographical region, climate, and soil conditions. Furthermore, the analysis of different *Salvia* species may have contributed to this difference.

In a previous study, cynaroside and rosmarinic acid were found to be the most abundant compounds in *S. balansae* Noë ex Coss. flower powder extract (3.8 and 3.145 mg/g extract, respectively). Furthermore, *S. balansae* has been found to contain fumaric acid, acacetin, quinic acid, luteolin, apigenin, protocatechuic acid, salicylic acid, protocatechuic aldehyde, and cosmosiin (Souidi et al. 2025). We similarly detected these compounds in all extracts (*S. vermifolia*, *S. ballsiana*, and *S. hedgeana*). Additionally, we found higher levels of rosmarinic acid in all extracts compared to *S. balansae* . Rosmarinic acid was found in the highest abundance in the aerial parts (ethanolic extract) of *S. macrochlamys* Boiss. & Kotschy (3919.65 mg/kg) (Kızıltaş et al. 2024). Similarly, rosmarinic acid was found to be the most abundant in the extracts of *S. blepharochlaena* Hedge & Hub.‐Mor., *S. euphratica* var. *leiocalycina* (Rech.f.) Hedge, and *S. verticillata* subsp. *amasiaca* (Freyn & Bornm.) Bornm. (ranged between 10 and 67 mg/g extract) (Zengin et al. 2018). In a study conducted by Yilmaz et al., the aerial parts of *S. aucheri* subsp. *canescens* (Boiss. & Heldr.) Celep, Kahraman & Doğan, *S*. *aytachii* Vural & Adıgüzel, *S. heldreichiana* Boiss. ex Benth., *S. viridis* L., and *S*. *wiedemannii* Boiss. were found to contain the most rosmarinic acid. Furthermore, caffeic acid, protocatechuic acid, cynaroside, protocatechuic aldehyde, cosmosiin, acacetin, rosmarinic acid, naringenin, apigenin, and luteolin were detected in all extracts (Yilmaz et al. 2023). Similar to the study by Yilmaz et al., we detected all of these compounds in extracts of *S. ballsiana*, *S. hedgeana*, and *S. vermifolia*. This can be explained by the fact that plant species belonging to the same genus have similar phytochemical components. Based on this information, we have revealed that the extracts of *S. ballsiana*, *S. hedgeana*, and *S. vermifolia* possess a rich chemical composition.

In the treatment of type II diabetes, α‐amylase and α‐glucosidase enzymes are important targets. Inhibition of these enzymes prevents the breakdown of carbohydrates and lowers blood glucose levels. However, because currently used synthetic enzyme inhibitors have side effects such as liver problems, lactic acidosis, diarrhea, and hypoglycemia, researching new sources of enzyme inhibitors has become an important issue (Dahiya et al. 2025; Febriyanti et al. 2025). In diabetes management, medicinal plants are safe and inexpensive sources for inhibiting these enzymes (Kashtoh and Baek 2023). In light of this information, we investigated the antidiabetic effects of *S. ballsiana*, *S. hedgeana*, and *S. vermifolia* using α‐amylase and α‐glucosidase enzyme inhibition methods. Extracts of *S. vermifolia* and *S. hedgeana* strongly inhibited α‐glucosidase, while the extract of *S. ballsiana* moderately inhibited the enzyme (IC_50_ values were 36.46, 52.89, and 129.06 μg/mL, respectively). Furthermore, all extracts inhibited α‐amylase, and the enzyme inhibition of the *S. vermifolia* extract was notable. It has been reported that extracts rich in rosmarinic acid exhibit strong α‐amylase and α‐glucosidase inhibitory activities (Ngo and Chua 2018). Mervic et al. evaluated the α‐glucosidase inhibitor activity of leaf extracts from the species *S. fruticosa* Mill., *S. glutinosa* L., *S. nemorosa*, *S. officinalis* , *S. pratensis* L., *S. sclarea*, and *S. verticillata* . When the results were examined, it was observed that the *S. officinalis* extract had the strongest activity on α‐glucosidase (IC_50_ value: 4451.85 μg/mL). Additionally, it was reported that this extract had the highest rosmarinic acid content (38.800 μg/g) (Mervic et al. 2022). Adımcılar et al. evaluated the antidiabetic effect of rosmarinic acid containing extracts of 14 *Salvia* species using the α‐glucosidase inhibition test. The α‐glucosidase IC_50_ values of these *Salvia* extracts were found to range from 17.6 to 173 μg/mL. The authors reported a strong correlation between the amount of rosmarinic acid in the extracts and their biological activity (Adimcilar et al. 2019). The results of these studies are consistent with our study. We found that the *S. vermifolia* extract, which has the highest rosmarinic acid content, showed the strongest inhibitory activities. A previous study evaluated the enzyme inhibitory activity of the roots and aerial parts of *S. dorystaechas*. The IC_50_ value of root ethyl acetate extract on α‐glucosidase was found to be 47.67 μg/mL, while the effect of the extract on α‐amylase was found to be weak. Furthermore, the extract prepared from the aerial parts was found to have weaker inhibition of α‐glucosidase and α‐amylase (% inhibition values 35.88% and 18.99%, respectively). Interestingly, the extract prepared from the aerial parts of the plant contained higher levels of rosmarinic acid compared to the root extract (4.866 and 3.164 mg/g extract, respectively). The results of this study differ from ours (Ozupek et al. 2025). This suggests that, in addition to rosmarinic acid, other phenolic compounds may also make a significant contribution to enzyme inhibitory activity. In another study, the aerial parts of *S. aurita* L.f. inhibited α‐glucosidase (IC_50_ = 241.9 μg/mL) but had no effect on α‐amylase. Interestingly, carnosol, rosmanol, and 12‐methoxycarnosic acid compounds isolated from *S. aurita* inhibited α‐amylase (19.8, 40.9, and 16.2 μg/mL, respectively). Similarly, these isolated compounds were found to have stronger α‐glucosidase inhibitory activity compared to the crude extract. This can be explained by the low concentrations of these compounds in the extract (Etsassala et al. 2020). Mamache et al. evaluated the α‐glucosidase and α‐amylase inhibitor activities of *S. aegyptiaca* L. and *S. verbenaca* L. Methanol extracts of the aerial parts of *S. aegyptiaca* and *S. verbenaca* inhibited α‐amylase (IC_50_ values were 86.12 and 101.30 μg/mL, respectively). The IC_50_ values of *S. aegyptiaca* and *S. verbenaca* extracts on α‐glucosidase were found to be 96.94 and 150.5 μg/mL, respectively. The fact that the *S. aegyptiaca* extract has a stronger α‐glucosidase and α‐amylase inhibitory effect can be explained by its higher total phenol and total flavonoid content (Mamache et al. 2020). Similarly to Mamache et al., we also found that the *S. vermifolia* extract, which had higher total phenol and total flavonoid contents, exhibited stronger α‐glucosidase and α‐amylase inhibitory activities compared to the other extracts. This study is the first to evaluate the α‐amylase and α‐glucosidase inhibition of *S. ballsiana*, *S. hedgeana*, and *S. vermifolia*. *S. vermifolia* has been shown to strongly inhibit α‐glucosidase and α‐amylase enzymes. LC–MS/MS analysis results showed that *S. vermifolia* was richer in fumaric acid, protocatechuic acid, protocatechuic aldehyde, caffeic acid, *p*‐coumaric acid, salicylic acid, genistin, rosmarinic acid, cosmosiin, luteolin, apigenin, and acacetin components compared to other extracts. In this context, the strong α‐amylase and α‐glucosidase inhibitory activity of *S. vermifolia* may be related to its rich chemical composition. At the same time, this suggests that rosmarinic acid, which is found in high amounts in the *S. vermifolia*, may have an effect on enzyme inhibition. Furthermore, this situation can be explained by the synergistic effect of the phenolic compounds in the extract.

Obesity, defined as excessive fat accumulation in the body, causes serious health problems (type 2 diabetes, hypertension, cardiovascular diseases, etc.). Pancreatic lipase inhibitors, used in the treatment of obesity, reduce lipid absorption (Shamarao and Chethankumar 2022). In this context, pancreatic lipase inhibition is an effective strategy in the treatment of obesity. Generally, natural sources exhibit pancreatic lipase inhibitory effects (Wang et al. 2022). To our knowledge, this study is the first to demonstrate the pancreatic lipase activity of *S. ballsiana*, *S. hedgeana*, and *S. vermifolia* extracts. The results of this study revealed that *S. vermifolia* extract has moderate pancreatic lipase inhibitory activity (IC_50_ = 89.46 ± 6.80 μg/mL). Additionally, *S. ballsiana* and *S. hedgeana* extracts were found to have a weak pancreatic lipase effect. We mentioned that *S. vermifolia* has a richer chemical composition compared to other extracts. Therefore, the major component rosmarinic acid, together with the other phytochemical components, may have contributed to the pancreatic lipase activity. Previous studies have shown that rosmarinic acid has pancreatic lipase inhibitory activity (Auti et al. 2025; Bustanji et al. 2010). Marrelli et al. (2021) found that *S. miltiorrhiza* Bunge extract inhibited pancreatic lipase in a concentration‐dependent manner, with an IC_50_ value 3.54 mg/mL. Ozupek et al. (2025) reported a weak pancreatic lipase inhibitory activity of the *S*. *dorystaechas* with an IC_50_ value of 232 μg/mL. Another study found that *S. triloba* extract inhibited pancreatic lipase by 22.79% (Özüpek et al. 2023). In this context, the fact that *S. vermifolia* extract exhibited stronger pancreatic lipase inhibition compared to previously evaluated *S. milthorrhiza*, *S. dorystaechas*, and *S. triloba* extracts may be related to the richness of the *S. vermifolia* extract in individual phenolic and flavonoid content.

Docking procedure was validated by re‐docking co‐crystalline acarbose to α‐amylase (2QV4) and α‐glucosidase (8YIE) structures. RMSD value of acarbose was detected to be below 2 Å, the upper threshold for a reliable docking (Muhammed and Aki‐Yalcin 2024). Together with this, acarbose's interaction profile in the docking study had a high level of similarity to reported studies. A previous crystallographic study reported acarbose's binding to the same α‐amylase structure (2QV4) via various amino acid residues including Thr163, His201, and His299 (Maurus et al. 2008). This means interaction residual similarity between docking and experimental study would be high as half of the residues are common. Similarly, a previous crystallographic study reported acarbose's interaction to the same α‐glucosidase structure (8YIE) through the involvement of various amino acid residues including Asp221, Glu278, and Asp337 (Saburi et al. 2024). All acarbose's interactions in the docking were observed in the experimental study showing an exact match between the two methods. In short, acarbose's interactions in the docking were confirmed by reported experimental studies in general. Therefore, parameter preferences were estimated to give reproducible results for rosmarinic acid too.

Docking study implied that rosmarinic acid could bind to α‐amylase, α‐glucosidase, and pancreatic lipase structures. Binding energy related values of rosmarinic acid in the interaction to the three enzyme structures were near to each other. This result indicates that binding affinity of rosmarinic acid would be like each other. Rosmarinic acid formed at least three H‐bonds with enzyme structures. Lower binding energy value for a ligand is correlated to higher binding affinity (Saleem et al. 2026). As a result, binding affinity of rosmarinic acid was found to be lower in comparison to acarbose (Table 6). In addition, the number of H‐bonds or other interactions formed by rosmarinic acid with α‐amylase and α‐glucosidase was lower or like acarbose (Figures 3 and 4). On the other hand, rosmarinic acid formed a higher number of interactions with pancreatic lipase (Figure 5). Similarly, binding energy related values for rosmarinic acid were lower in comparison to orlistat implying higher binding affinity (Table 6).

Rosmarinic acid could bind to α‐amylase and α‐glucosidase structures but lower than co‐crystalline acarbose. This was enacted from their lower binding affinity and lower number of interactions. This result is in line with in vitro enzymatic assay study results. In the experimental study, acarbose was more active than the most active extract on α‐amylase and α‐glucosidase. The probable binding potential of the extract was explored through docking of its major phytoconstituent, rosmarinic acid, on enzyme structures. Results of docking and experimental studies about the relative activity of acarbose and extract were found to be in harmony. On the other hand, rosmarinic acid was anticipated to have higher binding potential than orlistat on pancreatic lipase structure though the most active extract had less activity than orlistat in the enzymatic assay study. Previous studies reported the binding potential of orlistat on pancreatic lipase structures using docking. A study reported that orlistat had lower binding affinity to pancreatic lipase structure in comparison to some phthalimide‐linked 1,2,3‐triazoles. In this docking supported study, orlistat had a binding energy of −6.6 kcal/mol though synthesized compounds had values below −10 kcal/mol (Kizilkaya and Bursal 2026). This has implied lower binding affinity for orlistat. Another study supported with docking gave −8.7 kcal/mol energy for orlistat though phytoconstituent ligands gave values below nearly −11 kcal/mol in the interaction to human pancreatic lipase (Nishikawa et al. 2026). Hence, orlistat had lower binding affinity to human pancreatic lipase in previously reported studies too. The difference in binding energy related values between rosmarinic acid and orlistat was narrower in this study. In addition, higher binding affinity of rosmarinic acid in comparison to orlistat is in line with reported studies. The discrepancy in interaction results of orlistat could arise from its structure. Orlistat had long alkyl substituents. As a result, the long hydrophobic structures might hinder the binding of orlistat to the pancreatic lipase structure though the core structure contains favorable elements for ligand‐enzyme binding. Hence, the binding energy level of orlistat in this and previous studies might be a reflection of its structure.

Rosmarinic acid interacted with α‐amylase, α‐glucosidase, and pancreatic lipase structures according to docking results. Rosmarinic acid formed at least three H‐bonds with enzyme structures. Especially, the hydroxyl substituent of the two 3,4‐dihydroxyphenyls played a critical role in the formation of H‐bonds with enzyme structures. In this regard, three H‐bonds were formed with the involvement of these hydroxyl substituents in the interaction with α‐amylase and pancreatic lipase. Similarly, two H‐bonds were observed between the hydroxyl substituent and the α‐glucosidase structure. Carboxyl functional group of propanoic acid also played a role in the interaction of rosmarinic acid to α‐glucosidase and pancreatic lipase. In this regard, an H‐bond was formed with α‐glucosidase and an H‐bond as well as a salt bridge were formed with pancreatic lipase by involving this carboxyl group (Figures 3, 4, 5).

**FIGURE 6:**
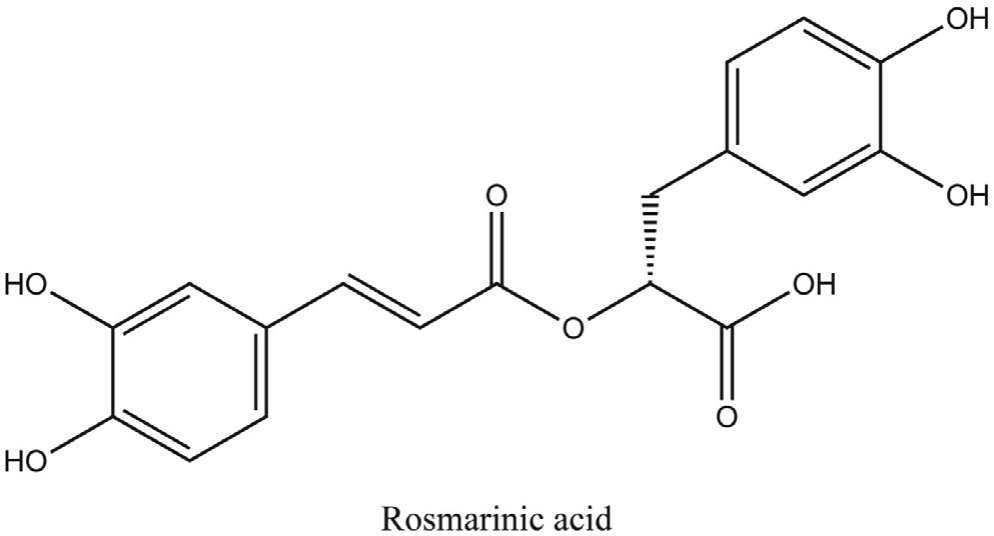
Chemical structure of rosmarinic acid.

Structure–activity relationship (SAR) analysis across the three enzyme structures reveals that rosmarinic acid is a multi‐targeting scaffold due to its ester‐linked catechols. In the interaction with α‐amylase, rosmarinic acid utilizes the flexibility of linker conformations to place its peripheral phenolic hydroxyl groups along the binding pocket. In the interaction with α‐glucosidase, rosmarinic acid displays two distinct modes in which the central carboxylic acid group and the left phenolic hydroxyl group serve as primary polar anchors. In the interaction with pancreatic lipase, rosmarinic acid exploits its central carboxylate anion and the terminal phenolic hydroxyl groups to form a robust salt bridge and multiple H‐bonds. Collectively, SAR analysis indicates that the spatial arrangement of the terminal phenolic hydroxyl groups, combined with the electrostatic pull of the core carboxylate moiety and flexibility of the ester linker, are drivers of its broad enzymatic potency (Figure 6).

To our knowledge, there are no previously conducted toxicity studies on *S. ballsiana*, *S. hedgeana*, and *S. vermifolia*. *S. scutellarioides* Kunth, *S. przewalskii* Maxim. and *S. chloroleuca* Rech.f. & Aellen have been reported to exhibit no significant toxic effects in in vivo experiments (Li et al. 2010; Ramirez et al. 2007; Salimikia et al. 2015). Despite these findings, it has been reported in the literature that *Salvia* species may exhibit cytotoxic effects due to their luteolin, apigenin, and chrysin contents, and that these species should be used with caution (Irtegun Kandemir et al. 2022).

This study has several limitations. Since the activities investigated in the study were evaluated using in vitro methods, the findings need to be supported by in vivo studies. The use of different solvents in extract preparation may lead to variations in the results. Furthermore, the phytochemical content of medicinal plants may vary depending on environmental and geographical factors, which may influence their biological activity. Future research should focus on in vivo studies, cellular level mechanistic investigations, and more advanced computational approaches. In addition, the isolation and characterization of bioactive compounds should be performed to elucidate the therapeutic potential of *Salvia* species.

## 5 Conclusions

This is the first report of α‐amylase, α‐glucosidase, and pancreatic lipase inhibition by *S. ballsiana*, *S. hedgeana*, and *S. vermifolia* extracts. LC–MS/MS analysis revealed rosmarinic acid as the main component in *S. vermifolia* and *S. hedgeana*. The extracts have been shown to possess notable antioxidant and antidiabetic activities. *S. vermifolia* extract, which is richest in rosmarinic acid, was found to have a stronger effect on all enzyme systems examined. Docking study disclosed that rosmarinic acid could bind to α‐amylase, α‐glucosidase, and pancreatic lipase structures. Together with this, binding potential of rosmarinic acid was found to be lower in comparison to acarbose. Future studies should in vivo evaluate the effects demonstrated by *S. vermifolia*, with a focus on toxicity and pharmacokinetic studies, as well as the isolation of the active compounds present in this plant.

## Author Contributions

**Semih Bulut:** conceptualization, methodology, data curation, investigation, formal analysis, supervision, visualization, project administration, writing – original draft, writing – review and editing. **Sümeyye Usta:** data curation, investigation, visualization, writing – original draft, writing – review and editing, methodology, formal analysis. **Ahmet Kahraman:** methodology, investigation, visualization, writing – original draft, writing – review and editing, data curation. **Mustafa Abdullah Yilmaz:** methodology, data curation, validation, writing – original draft, writing – review and editing. **Oguz Cakir:** data curation, writing – review and editing, writing – original draft, methodology. **Muhammed Tilahun Muhammed:** investigation, writing – original draft, writing – review and editing, data curation, methodology.

## Funding

The authors thank the Scientific Research Projects Coordination Unit of Suleyman Demirel University for financial support (Project number: TMA‐2025‐9788).

## Supporting information


**Table S1:** Validation parameters for the standard compounds.

## Data Availability

The data that support the findings of this study are available from the corresponding author upon reasonable request.
