# Supplementary material for: Phytochemical Profiling and Evaluation of Antioxidant and Enzyme Inhibitory Activities of Selected Endemic Salvia Species (Lamiaceae) From Türkiye
Source: Food Sci Nutr. 2026 Jul 22;14(7):e72149. doi: 10.1002/fsn3.72149 (PMC13392212; doi:10.1002/fsn3.72149)
Supplement: Supplementary file 1 — Table S1: Validation parameters for the standard compounds. [file FSN3-14-e72149-s001.pdf]

Table S1. Validation parameters for the standard compounds

No	Analytes	RT[a]	M.I. (m/z)[b]	F.I. (m/z)[c]	Ion. mode	Equation	r^{2d}	*RSD*%[e]		Linearity Range (mg/L)	*LOD*/*LOQ* (µg/L)[f]	Recovery (%)		*U*[g]	Gr. No[i]
								Interday	Intraday			Interday	Intraday		
1	Quinic acid	3.0	190.8	93.0	Neg	*y*=-0.0129989+2.97989×	0.996	0.69	0.51	0.1-5	25.7/33.3	1.0011	1.0083	0.0372	1
2	Fumaric aid	3.9	115.2	40.9	Neg	*y*=-0.0817862+1.03467×	0.995	1.05	1.02	1-50	135.7/167.9	0.9963	1.0016	0.0091	1
3	Aconitic acid	4.0	172.8	129.0	Neg	*y*=-0.7014530+32.9994×	0.971	2.07	0.93	0.1-5	16.4/31.4	0.9968	1.0068	0.0247	1
4	Gallic acid	4.4	168.8	79.0	Neg	*y*=0.0547697+20.8152×	0.999	1.60	0.81	0.1-5	13.2/17.0	1.0010	0.9947	0.0112	1
5	Epigallocatechin	6.7	304.8	219.0	Neg	*y*=-0.00494986+0.0483704×	0.998	1.22	0.73	1-50	237.5/265.9	0.9969	1.0040	0.0184	3
6	Protocatechuic acid	6.8	152.8	108.0	Neg	*y*=0.211373+12.8622×	0.957	1.43	0.76	0.1-5	21.9/38.6	0.9972	1.0055	0.0350	1
7	Catechin	7.4	288.8	203.1	Neg	*y*=-0.00370053+0.431369×	0.999	2.14	1.08	0.2-10	55.0/78.0	1.0024	1.0045	0.0221	3
8	Gentisic acid	8.3	152.8	109.0	Neg	*y*=-0.0238983+12.1494×	0.997	1.81	1.22	0.1-5	18.5/28.2	0.9963	1.0077	0.0167	1
9	Chlorogenic acid	8.4	353.0	85.0	Neg	*y*=0.289983+36.3926×	0.995	2.15	1.52	0.1-5	13.1/17.6	1.0000	1.0023	0.0213	1
10	Protocatechuic aldehyde	8.5	137.2	92.0	Neg	*y*=0.257085+25.4657×	0.996	2.08	0.57	0.1-5	15.4/22.2	1.0002	0.9988	0.0396	1
11	Tannic acid	9.2	182.8	78.0	Neg	*y*=0.0126307+26.9263×	0.999	2.40	1.16	0.05-2.5	15.3/22.7	0.9970	0.9950	0.0190	1
12	Epigallocatechin gallate	9.4	457.0	305.1	Neg	*y*=-0.0380744+1.61233×	0.999	1.30	0.63	0.2-10	61.0/86.0	0.9981	1.0079	0.0147	3
13	1,5-dicaffeoylquinic acid	9.8	515.0	191.0	Neg	*y*=-0.0164044+16.6535×	0.999	2.42	1.48	0.1-5	5.8/9.4	0.9983	0.9997	0.0306	1
14	4-OH Benzoic acid	10.5	137.2	65.0	Neg	*y*=-0.0240747+5.06492×	0.999	1.24	0.97	0.2-10	68.4/88.1	1.0032	1.0068	0.0237	1
15	Epicatechin	11.6	289.0	203.0	Neg	*y*=-0.0172078+0.0833424×	0.996	1.47	0.62	1-50	139.6/161.6	1.0013	1.0012	0.0221	3
16	Vanilic acid	11.8	166.8	108.0	Neg	*y*=-0.0480183+0.779564×	0.999	1.92	0.76	1-50	141.9/164.9	1.0022	0.9998	0.0145	1
17	Caffeic acid	12.1	179.0	134.0	Neg	*y*=0.120319+95.4610×	0.999	1.11	1.25	0.05-2.5	7.7/9.5	1.0015	1.0042	0.0152	1
18	Syringic acid	12.6	196.8	166.9	Neg	*y*=-0.0458599+0.663948×	0.998	1.18	1.09	1-50	82.3/104.5	1.0006	1.0072	0.0129	1
19	Vanillin	13.9	153.1	125.0	Poz	*y*=0.00185898+20.7382×	0.996	1.10	0.85	0.1-5	24.5/30.4	1.0009	0.9967	0.0122	1
20	Syringic aldehyde	14.6	181.0	151.1	Neg	*y*=-0.0128684+7.90153×	0.999	2.51	0.77	0.4-20	19.7/28.0	1.0001	0.9964	0.0215	1
21	Daidzin	15.2	417.1	199.0	Poz	*y*=9.45747+152.338×	0.996	2.25	1.32	0.05-2.5	7.0/9.5	0.9955	1.0017	0.0202	2
22	Epicatechin gallate	15.5	441.0	289.0	Neg	*y*=-0.0142216+1.06768×	0.997	1.63	1.28	0.1-5	19.5/28.5	0.9984	0.9946	0.0229	3
23	Piceid	17.2	391.0	135/106.9	Poz	*y*=0.00772525+25.4181×	0.999	1.94	1.16	0.05-2.5	13.8/17.8	1.0042	0.9979	0.0199	1
24	*p*-Coumaric acid	17.8	163.0	93.0	Neg	*y*=0.0249034+18.5180×	0.999	1.92	1.43	0.1-5	25.9/34.9	1.0049	1.0001	0.0194	1
25	Ferulic acid-D3-IS[h]	18.8	196.2	152.1	Neg	N.A.	N.A.	N.A.	N.A.	N.A.	N.A.	N.A.	N.A.	0.0170	1
26	Ferulic acid	18.8	192.8	149.0	Neg	*y*=-0.0735254+1.34476×	0.999	1.44	0.53	1-50	11.8/15.6	0.9951	0.9976	0.0181	1
27	Sinapic acid	18.9	222.8	193.0	Neg	*y*=-0.0929932+0.836324×	0.999	1.45	0.52	0.2-10	65.2/82.3	1.0031	1.0037	0.0317	1
28	Coumarin	20.9	146.9	103.1	Poz	*y*=0.0633397+136.508×	0.999	2.11	1.54	0.05-2.5	214.2/247.3	0.9950	0.9958	0.0383	1

[a]R.T.: Retention time, [b]MI *(m/z):* Molecular ions of the standard analytes (m/z ratio), [c]FI *(m/z):* Fragment ions [d]r^2: Coefficient of determination, [e]*RSD*: Relative standard deviation, [f]*LOD*/*LOQ* (µg/L): Limit of detection/quantification, [g]*U* (%): percent relative uncertainty at 95% confidence level (k = 2), [h]IS: Internal standard, [i]Gr. No: Represents grouping of internal standards, these numbers indicate which IS stands for which phenolic compound.

Table S1. Validation parameters for the standard compounds (Continued)

No	Analytes	RT[a]	M.I. (m/z)[b]	F.I. (m/z)[c]	Ion. mode	Equation	r^{2d}	*RSD%*[e]		Linearity Range (mg/L)	*LOD*/*LOQ* (µg/L)[f]	Recovery (%)		*U*[g]	Gr. No
								Interday	Intraday			Interday	Intraday		
29	Salicylic acid	21.8	137.2	65.0	Neg	*y*=0.239287+153.659×	0.999	1.48	1.18	0.05-2.5	6.0/8.3	0.9950	0.9998	0.0158	1
30	Cynaroside	23.7	447.0	284.0	Neg	*y*=0.280246+6.13360×	0.997	1.56	1.12	0.05-2.5	12.1/16.0	1.0072	1.0002	0.0366	2
31	Miquelianin	24.1	477.0	150.9	Neg	*y*=-0.00991585+5.50334×	0.999	1.31	0.95	0.1-5	10.6/14.7	0.9934	0.9965	0.0220	2
32	Rutin-D3-IS[h]	25.5	612.2	304.1	Neg	N.A.	N.A.	N.A.	N.A.	N.A.	N.A.	N.A.	N.A.	N.A.	2
33	Rutin	25.6	608.9	301.0	Neg	*y*=-0.0771907+2.89868×	0.999	1.38	1.09	0.1-5	15.7/22.7	0.9977	1.0033	0.0247	2
34	isoquercitrin	25.6	463.0	271.0	Neg	*y*=-0.111120+4.10546×	0.998	2.13	0.78	0.1-5	8.7/13.5	1.0057	0.9963	0.0220	2
35	Hesperidin	25.8	611.2	449.0	Poz	*y*=0.139055+13.2785×	0.999	1.84	1.35	0.1-5	19.0/26.0	0.9967	1.0043	0.0335	2
36	*o*-Coumaric acid	26.1	162.8	93.0	Neg	*y*=0.00837193+11.2147×	0.999	2.11	1.46	0.1-5	31.8/40.4	1.0044	0.9986	0.0147	1
37	Genistin	26.3	431.0	239.0	Neg	*y*=1.65808+7.57459×	0.991	2.01	1.28	0.1-5	14.9/21.7	1.0062	1.0047	0.0083	2
38	Rosmarinic acid	26.6	359.0	197.0	Neg	*y*=-0.0117238+8.04377×	0.999	1.24	0.86	0.1-5	16.2/21.2	1.0056	1.0002	0.0130	1
39	Ellagic acid	27.6	301.0	284.0	Neg	*y*=0.00877034+0.663741×	0.999	1.57	1.23	0.4-20	56.9/71.0	1.0005	1.0048	0.0364	1
40	Cosmosiin	28.2	431.0	269.0	Neg	*y*=-0.708662+8.62498×	0.998	1.65	1.30	0.1-5	6.3/9.2	0.9940	0.9973	0.0083	2
41	Quercitrin	29.8	447.0	301.0	Neg	*y*=-0.00153274+3.20368×	0.999	2.24	1.16	0.1-5	4.8/6.4	0.9960	0.9978	0.0268	2
42	Astragalin	30.4	447.0	255.0	Neg	*y*=0.00825333+3.51189×	0.999	2.08	1.72	0.1-5	6.6/8.2	0.9968	0.9957	0.0114	2
43	Nicotiflorin	30.6	592.9	255.0/284.0	Neg	*y*=0.00499333+2.62351×	0.999	1.48	1.23	0.05-2.5	11.9/16.7	0.9954	1.0044	0.0108	2
44	Fisetin	30.6	285.0	163.0	Neg	*y*=0.0365705+8.09472×	0.999	1.75	1.19	0.1-5	10.1/12.7	0.9980	1.0042	0.0231	3
45	Daidzein	34.0	253.0	223.0	Neg	*y*=-0.0329252+6.23004×	0.999	2.18	1.73	0.1-5	9.8/11.6	0.9926	0.9963	0.0370	3
46	Quercetin-D3-IS[h]	35.6	304.0	275.9	Neg	N.A.	N.A.	N.A.	N.A.	N.A.	N.A.	N.A.	N.A.	N.A.	3
47	Quercetin	35.7	301.0	272.9	Neg	*y*=+0.00597342+3.39417×	0.999	1.89	1.38	0.1-5	15.5/19.0	0.9967	0.9971	0.0175	3
48	Naringenin	35.9	270.9	119.0	Neg	*y*=-0.00393403+14.6424×	0.999	2.34	1.69	0.1-5	2.6/3.9	1.0062	1.0020	0.0392	3
49	Hesperetin	36.7	301.0	136.0/286.0	Neg	*y*=+0.0442350+6.07160×	0.999	2.47	2.13	0.1-5	7.1/9.1	0.9998	0.9963	0.0321	3
50	Luteolin	36.7	284.8	151.0/175.0	Neg	*y*=-0.0541723+30.7422×	0.999	1.67	1.28	0.05-2.5	2.6/4.1	0.9952	1.0029	0.0313	3
51	Genistein	36.9	269.0	135.0	Neg	*y*=-0.00507501+12.1933×	0.999	1.48	1.19	0.05-2.5	3.7/5.3	1.0069	1.0012	0.0337	3
52	Kaempferol	37.9	285.0	239.0	Neg	*y*=-0.00459557+3.13754×	0.999	1.49	1.26	0.05-2.5	10.2/15.4	0.9992	0.9990	0.0212	3
53	Apigenin	38.2	268.8	151.0/149.0	Neg	*y*=0.119018+34.8730×	0.998	1.17	0.96	0.05-2.5	1.3/2.0	0.9985	1.0003	0.0178	3
54	Amentoflavone	39.7	537.0	417.0	Neg	*y*=0.727280+33.3658×	0.992	1.35	1.12	0.05-2.5	2.8/5.1	0.9991	1.0044	0.0340	3
55	Chrysin	40.5	252.8	145.0/119.0	Neg	*y*=-0.0777300+18.8873×	0.999	1.46	1.21	0.05-2.5	1.5/2.8	0.9922	1.0050	0.0323	3
56	Acacetin	40.7	283.0	239.0	Neg	*y*=-0.559818+163.062×	0.997	1.67	1.28	0.02-1	1.5/2.5	0.9949	1.0011	0.0363	3

[a]R.T.: Retention time, [b]MI (*m/z):* Molecular ions of the standard analytes (m/z ratio), [c]FI (*m/z):* Fragment ions [d]r^2: Coefficient of determination, [e]*RSD*: Relative standard deviation, [f]*LOD*/*LOQ* (µg/L): Limit of detection/quantification, [g]*U* (%): percent relative uncertainty at 95% confidence level (k = 2), [h]IS: Internal standard, [i]Gr. No: Represents grouping of internal standards, these numbers indicate which IS stands for which phenolic compound.
